# Distinct miRNA expression patterns in circulating eosinophil subtypes and their subtype-derived exosomes in allergic asthma

**DOI:** 10.1007/s00011-026-02348-w

**Published:** 2026-08-18

**Authors:** Egle Vasyle, Andrius Januskevicius, Airidas Rimkunas, Jolita Palacionyte, Skaidrius Miliauskas, Kestutis Malakauskas

**Affiliations:** 1 Laboratory of Pulmonology, Department of Pulmonology, Lithuanian University of Health Sciences, A. Mickeviciaus St. 9, LT-44307 Kaunas, Lithuania; 2 Department of Pulmonology, Lithuanian University of Health Sciences, Kaunas, Lithuania

**Keywords:** Asthma, Exosomes, Inflammatory eosinophil, Lung-resident eosinophil, miRNA

## Abstract

**Background:**

Lung-resident (rEos) and inflammatory (iEos) eosinophil subtypes were initially characterized in mouse models of asthma and have been proposed to contribute differentially to inflammation and remodeling in human allergic asthma (AA). While microRNAs (miRNAs) and their transfer through exosomes may play important regulatory roles in immune signaling in asthma, miRNA profiles specific to eosinophil subtypes remain uncharacterized. This study aimed to identify distinct miRNA expression patterns in circulating eosinophil subtypes and subtype-derived exosomes from AA patients.

**Methods:**

Peripheral blood iEos and rEos were isolated from 22 AA patients and 21 healthy subjects (HS). Next-generation sequencing (NGS) was used to identify differentially expressed miRNAs, and 16 candidates were subsequently selected for validation by quantitative polymerase chain reaction (qPCR) in eosinophil subtypes and in subtype-derived exosomes.

**Results:**

NGS revealed 62 differentially expressed miRNAs in AA iEos and 35 in AA rEos compared with corresponding HS eosinophil subtypes. qPCR confirmed differential expression for 14 of 16 selected miRNAs in eosinophils or their exosomes between AA and HS. miRNA expressions distinguished AA from HS; furthermore, iEos and rEos showed distinct miRNA patterns within the AA group. Notably, AA rEos-derived exosomes showed greater miRNA enrichment than iEos-derived exosomes, implying active exosomal communication. Finally, validated miRNA patterns suggest a predominance of regulatory functions in rEos, whereas iEos displayed a profile consistent with pro-inflammatory activation.

**Conclusions:**

This study provides molecular evidence that eosinophil subtypes are functionally specialized in AA. The differential miRNA patterns of rEos and iEos highlight their complementary regulatory and pro-inflammatory roles and suggest that eosinophil-derived miRNAs merit further investigation as potential biomarkers or modulators of airway inflammation.

**Supplementary Information:**

The online version contains supplementary material available at 10.1007/s00011-026-02348-w.

## Introduction

Asthma comprises several immunologically distinct endotypes, among which type 2 (T2) AA represents one of the most prevalent chronic inflammatory airway diseases worldwide. T helper 2 (Th2) cells are central drivers of this phenotype, producing interleukins (IL)-4, IL-5, and IL-13 that collectively promote eosinophil recruitment, activation, and prolonged survival within the lungs. Eosinophils have long been recognized as key effector cells in allergic inflammation, contributing to airway remodeling, mucus hypersecretion, and disease exacerbation. These functions are mediated through the release of multiple inflammatory mediators, chemokines, lipid-derived molecules and cytoplasmic granules containing cytotoxic proteins [1, 2].

Recent findings suggest a more nuanced role for eosinophils, with the identification of distinct subtypes exhibiting potentially distinct functions in the context of asthma [3–8]. These subtypes, rEos and iEos, differ in their gene expression profiles and functional properties. rEos are primarily associated with tissue homeostasis, repair, antimicrobial defense, and downregulation of Th2 responses, whereas iEos exhibit a more pro-inflammatory phenotype characterized by the expression of inflammation-related genes and by cytolytic degranulation, which leads to the massive release of granule proteins, tissue damage, and prolonged inflammation [3]. Consequently, these subtypes are hypothesized to contribute differently to the initiation, maintenance, and resolution of airway inflammation. Elucidating the molecular mechanisms underlying the specialized activities of these subtypes is therefore essential for the development of more precise therapeutic approaches.

miRNAs are small, non-coding RNAs of approximately 22 nucleotides that regulate gene expression at the post-transcriptional level. By binding to complementary sequences in the 3′ untranslated region (UTR) of target messenger RNAs (mRNAs), they induce mRNA degradation or translational repression, thereby fine-tuning diverse cellular processes. Dysregulation of miRNA expression has been implicated in the pathogenesis of numerous diseases, including asthma [9–12]. These regulatory molecules influence the development, differentiation, and effector functions of immune cells, and altered miRNA profiles have been linked to asthma severity, the risk of exacerbation, and therapeutic response [9, 13–15]. In addition, miRNAs can be selectively packaged and secreted via extracellular vesicles, particularly exosomes, which serve as carriers of molecular cargo (including proteins, lipids, nucleic acids, and miRNAs) mediating intercellular communication in both physiological and pathological context [16, 17]. In asthma, exosomes released by immune and structural cells of the lung contribute to inflammatory regulation [18–20], and those from asthmatic patients differ from healthy individuals in abundance, molecular composition, and biological activity [21]. Eosinophils, in particular, have been shown to release higher numbers of exosomes in asthma [22], and these eosinophil-derived exosomes can further modulate the function of neighboring immune and airway structural cells, amplifying inflammation and remodeling through autocrine and paracrine signaling [23]. Therefore, analyzing miRNA expressions not only within eosinophil subtypes but also in their exosomes may provide a more integrated understanding of eosinophil-mediated regulatory networks in asthma.

In this study, we identified differentially expressed miRNAs between rEos and iEos in patients with AA. Our central hypothesis is that distinct miRNA expression patterns exist between eosinophil subtypes their derived exosomes, contributing to their divergent roles in asthma pathogenesis. By characterizing and validating these profiles, we aim to advance understanding of the regulatory mechanisms underlying eosinophil heterogeneity and identify potential biomarkers or therapeutic targets.

## Materials and methods

### Study population

A total of 22 subjects with AA and 21 HS were recruited from the Department of Pulmonology at the Lithuanian University of Health Sciences, Kaunas Clinics. The diagnosis of AA was established based on asthma-related symptoms persisting for more than one year together with documented airway hyperresponsiveness to methacholine. Eligible patients had non-severe asthma classified as corresponding to GINA treatment Steps 1–2 and confirmed allergic sensitization to *Dermatophagoides pteronyssinus*. To minimize potential confounding related to corticosteroid exposure, only patients who had not used inhaled corticosteroids for at least one month before enrollment were included. The HS consisted of volunteers without a history of pulmonary diseases or allergies. All study participants were non-smokers. A detailed list of inclusion and exclusion criteria for both groups is provided in Supplementary Table S1.

After the inclusion and exclusion criteria were approved, eligible participants were informed about the study and invited to take part. All individuals were provided with adequate time to review the study information and gave written informed consent prior to the initiation of any procedures. Participants attended two hospital visits: an initial screening visit and a subsequent experimental session. A schematic overview of the procedures performed at each visit is presented in Supplementary Table S2.

### Ethics approval and participant consent

The study protocol was approved by the Regional Biomedical Ethics Committee (approval No. P3-BE-2-58/2020). Written informed consent was obtained from all participants prior to their enrollment in the study. The study adheres to the principles outlined in the Declaration of Helsinki. To maintain confidentiality, each participant was assigned a unique identification code. The study is registered on ClinicalTrials.gov (ID: NCT04542902).

### Skin prick testing

Allergy skin prick testing was performed using a panel of standardized extracts (Stallergenes S.A., France), which included cat and dog dander, birch pollen, a mixture of five grass pollens, mugwort, *Dermatophagoides pteronyssinus*, *Dermatophagoides farinae*, *Aspergillus*, *Alternaria*, and *Cladosporium*. Saline served as negative control, while histamine hydrochloride (10 mg/mL) was used as the positive control. Test results were assessed 15 min after application, and a wheal diameter greater than 3 mm was considered indicative of sensitization. Only AA patients with confirmed reactivity to *Dermatophagoides pteronyssinus* were included in the study.

### Pulmonary function evaluation

#### Spirometric assessment

Spirometry was performed in all study participants, with a minimum of three technically acceptable maneuvers required from each individual. Lung function measurements were obtained using an ultrasonic spirometer (Ganshorn Medizin Electronic, Niederlauer, Germany). The detailed methodology of spirometry is described elsewhere [8].

#### Methacholine challenge test

Participants in the AA group who did not exhibit airflow limitation at screening underwent a methacholine challenge test. The procedure was performed using a ProvoX dosimeter (Ganshorn Medizin Electronic, Niederlauer, Germany). Lung function was assessed every two minutes after methacholine inhalation, and the test was terminated upon reaching a 20% decline in FEV₁. The detailed protocol has been described previously [8].

#### FeNO measurement

Fractional exhaled nitric oxide (Fe_NO_) was measured in all study participants. Measurements were obtained using the Vivatmo-me device (Bosch Healthcare Solutions, Waiblingen, Germany) in accordance with the manufacturer’s instructions. Each evaluation was carried out at least twice to ensure accuracy.

### Peripheral blood count and immunoglobulin E

Peripheral blood samples were collected into dipotassium ethylenediaminetetraacetic acid (K_2_EDTA)-coated vacutainers (BD Vacutainer®, Becton Dickinson UK Ltd., Wokingham, UK). Complete blood counts were performed using an automated hematology analyzer XE-5000™ (Sysmex, Kobe, Japan), while serum immunoglobulin E (IgE) levels were determined with the automated immunoassay analyzer AIA-2000 (Tosoh Bioscience, South San Francisco, CA, USA).

### Isolation of eosinophils from peripheral blood and eosinophil subtyping

**Fig. 1:**
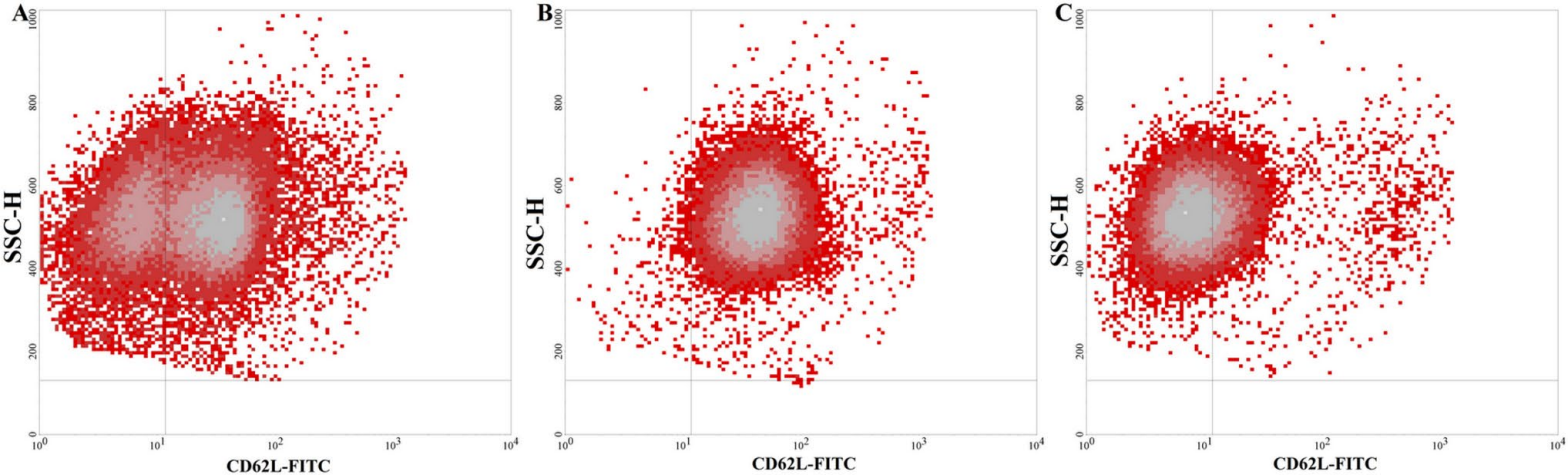
Representative flow-cytometry density plots of CD62L expression in blood eosinophils before and after magnetic separation. **A** Total eosinophils before separation, showing a heterogeneous and partially overlapping continuum of CD62L expression. The vertical line indicates the analysis threshold used to distinguish CD62L-low (left-side) and CD62L-high (right-side) eosinophils. **B** isolated rEos population after CD62L-FITC staining. **C** isolated iEos population after CD62L-FITC staining. Cell debris was excluded and population gated using a fixed threshold. iEos, inflammatory eosinophils; rEos, lung-resident eosinophils

Approximately 30 mL of peripheral blood was collected from each participant into K₂EDTA-coated vacutainers. Eosinophils were isolated and subtyped using magnetic beads and antibodies against CD62L (Miltenyi Biotec, Somerville, MA, USA). An adhesion molecule CD62L is predominantly expressed on rEOS but absent or expressed at low levels on iEOS [3]. Cells expressing sufficient levels of CD62L bound the magnetic beads and were retained in the column; after elution, these cells constituted the rEos-enriched fraction. Cells with low or undetectable CD62L expression did not bind sufficient numbers of magnetic beads, passed through the column, and constituted the iEos-enriched fraction. Eosinophil purity and viability were assessed with a FACS Calibur flow cytometer (BD, Franklin Lakes, NJ, USA) and an ADAM automated cell counter (NanoEnTek Inc., Mountain View, CA, USA), respectively. The eosinophil isolation kit used provides greater than 96% purity while maintaining high cell viability. A detailed description of the methodology is provided in [8].The confirmation of blood eosinophil subtypes was performed by CD62L-FITC staining (Fig. 1).

### Exosome isolation from eosinophil subtypes

Eosinophil subtypes were cultured in RPMI 1640 medium (without phenol red) supplemented with 10% exosome-depleted fetal bovine serum, 100 U/mL penicillin, 100 μg/mL streptomycin, 2.5 μg/mL amphotericin B (Fungizone), and cytokines IL-5 (5 ng/mL) and GM-CSF (10 ng/mL) for 24 h at 37 °C in a humidified incubator with 5% CO_2_ to maintain eosinophil viability and functional competence during ex vivo incubation, allowing exosomes to accumulate in the conditioned medium [24, 25]. Subsequently, IFN-γ was added at a final concentration of 10 ng/mL for 1 h as a final short-term activating stimulus, based on previous evidence that IFN-γ enhances exosome release from human eosinophils [22]. Because eosinophil subtyping resulted in a limited cell input, the 1 h exposure was selected as a pragmatic protocol adaptation after a preliminary feasibility check indicated that eosinophil viability was maintained under these conditions. Identical culture and stimulation conditions were applied to all eosinophil subtypes and study groups. Following stimulation, culture supernatants were collected and centrifuged at 2000 × g for 30 min at room temperature to remove cells and debris. The supernatant was collected, resuspended and filtered through a membrane with 0.22 μm-sized pores. The cleared supernatants were transferred to new tubes and mixed with the Total Exosome Isolation reagent (Thermo Fisher Scientific, USA) at a 2:1 ratio (supernatant:reagent). Samples were gently resuspended and incubated overnight at 4 °C. Exosomes were then pelleted by centrifugation at 10,000 × g for 1 h at 4 °C, resuspended in phosphate-buffered saline (PBS), and stored at –80 °C until further use.

### RNA isolation from eosinophil subtypes and subtype-derived exosomes

Total RNA, including small RNAs, was extracted from eosinophil subtypes and their respective exosomes following cell lysis and homogenization using the miRNeasy Micro Kit (Qiagen, Hilden, Germany). All procedures were performed in accordance with the manufacturer’s protocol to ensure RNA integrity and yield, which are critical for accurate miRNA expression analysis. Purified RNA samples were stored at − 80 °C until further use.

### Extracted RNA quality assessment

The concentration and quality of total RNA were evaluated using a Qubit 4 Fluorometer (Thermo Fisher Scientific, USA).

### Small RNA library preparation and next-generation sequencing

Small RNA libraries were prepared from total RNA isolated from eosinophil subtypes using the NEXTFLEX® Small RNA-Seq Kit v4 with unique dual indices (PerkinElmer, Austin, TX, USA), according to the manufacturer’s protocol. Library quality and concentration were assessed using a Qubit 4 Fluorometer and an Agilent TapeStation (Agilent Technologies, USA). Sequencing was performed on an Illumina NextSeq 500/550 platform using the High Output Kit v2.5 [75 cycles] in single-end mode (SE75), generating approximately 12 million reads per sample (~ 25 M reads per library on average).

### Small RNA sequencing data processing and analysis

Raw sequencing reads in FASTQ format were subjected to quality control, including removal of adapter sequences, reads shorter than 18 nt or longer than 30 nt, and low-quality bases with Phred scores < Q30. Clean reads were aligned to the human reference genome (GRCh38) using Bowtie, after filtering against Silva, GtRNAdb, Rfam, and Repbase databases to exclude rRNA, tRNA, snRNA, snoRNA, and repetitive elements. Known miRNAs were annotated based on miRBase v22.

Differential expression analysis of miRNAs was performed using the DESeq2 package [26], with *p*-values adjusted for multiple testing using the Benjamini–Hochberg procedure. As the NGS analysis was exploratory and aimed at identifying candidate miRNAs for subsequent RT-qPCR validation, an FDR threshold of < 0.10 was applied. To ensure that the identified differences were also of sufficient magnitude, miRNAs were considered differentially expressed when they met both criteria: FDR < 0.10 and an absolute log_2_ fold change (|log_2_(FC)|) ≥ 0.585, corresponding to a change of at least 1.5-fold in either direction.

Principal Component Analysis (PCA) and unsupervised hierarchical clustering were performed in R. The expression matrix was log-transformed using ln(x + 1) and cleaned by excluding entries with missing values before analysis. PCA was performed on the cleaned log-transformed expression matrix using the prcomp function without additional scaling and was visualized using the ggplot2 package. For heatmap visualization and hierarchical clustering only, miRNA expression values were row-standardized using Z-scores. Hierarchical clustering was performed using Pearson correlation-based distance and average linkage with the pheatmap package. Cluster stability was evaluated by multiscale bootstrap resampling with 1000 bootstrap replications using the pvclust package, with approximately unbiased (AU) values ≥ 95% considered to indicate strong support for a cluster.

To formally assess differences in overall miRNA expression profiles among the four groups, pairwise PERMANOVA was performed using Euclidean distances calculated from the same cleaned log-transformed expression matrix with the adonis2 function in the R package vegan. Comparisons between iEos and rEos within the AA and HS groups were performed using restricted permutations stratified by participant ID to account for paired samples, whereas AA and HS comparisons were treated as unpaired. Homogeneity of multivariate dispersion was assessed using betadisper followed by permutest. *P*-values from the six pairwise comparisons were adjusted for multiple testing using the Benjamini–Hochberg procedure.

### qPCR validation analysis

Total RNA isolated from eosinophil subtypes and their respective exosomes was reverse transcribed using the TaqMan™ MicroRNA Reverse Transcription Kit and miRNA-specific stem-loop primers from the TaqMan MicroRNA Assays (Applied Biosystems, Thermo Fisher Scientific, USA) for the following targets: hsa-miR-126-5p (ID 000451), hsa-miR-1287-5p (ID 002828), hsa-miR-339-3p (ID 002184), hsa-miR-365a-3p (ID 001020), hsa-miR-151a-5p (ID 002642), hsa-miR-23c (ID 463068_mat), hsa-miR-629-5p (ID 002436), hsa-miR-2115-3p (ID 241073_mat), hsa-let-7i-5p (ID 002221), hsa-miR-4286 (ID 241500_mat), hsa-miR-766-3p (ID 001986), hsa-miR-22-5p (ID 002301), hsa-miR-142-3p (ID 000464), hsa-let-7c-5p (ID 000379), hsa-miR-486-5p (ID 001278), hsa-let-7b-3p (ID 002404). Reverse transcription was carried out according to the manufacturer’s instructions.

qPCR was carried out using the TaqMan™ Universal Master Mix II, no UNG, and the corresponding TaqMan MicroRNA Assays on a 7500 Fast Real-Time PCR System (Applied Biosystems, Foster City, CA, USA). Each reaction was performed in triplicate. Cycling conditions were set according to the manufacturer’s protocol: initial enzyme activation at 95 °C for 10 min, followed by 40 cycles of denaturation at 95 °C for 15 s and annealing/extension at 60 °C for 1 min.

### miRNA expression and statistical analysis

qPCR data were normalized using the global mean method, where ΔCt values were obtained by subtracting the sample-specific mean Ct across all reliably detected miRNAs from the target Ct [27]. HS served as the reference group, and results are presented as log_2_(FC) values, reflecting fold changes relative to HS baseline expression levels (equivalent to − ΔΔCt).

Statistical significance between AA and HS was assessed using the Wilcoxon signed rank test, comparing log₂(FC) values to the hypothetical median of zero. Additional comparisons of miRNA expressions between eosinophil subtypes (iEos vs rEos) were performed within the AA group using the Wilcoxon signed rank test. A *p*-value < 0.05 was considered statistically significant.

## Results

### Study subject characteristics

Our study included 22 patients with AA and 21 HS. Compared with HS, the AA group exhibited significantly higher blood eosinophil counts, elevated Fe_NO_ levels, and increased total IgE concentrations (*p* < 0.05). In addition, AA patients showed significantly lower forced expiratory volume in 1 s (FEV_1_) (L) and percentage of predicted FEV_1_ (*p* < 0.05). Demographic and clinical characteristics of the study population are presented in Table 1.

**Table 1:** The demographic and clinical characteristics of the study population

Characteristic	AA	HS
Number, n	22	21
Sex, M/F	9/13	11/10
Age, years	28.2 ± 3.2	29.6 ± 1.1
BMI, kg/m^2^	25.20 ± 1.42	22.72 ± 1.30
Blood Eos count, × 10^9^/L	0.37 ± 0.05 *	0.12 ± 0.01
Total IgE, IU/mL	219.20 ± 56.12*	8.39 ± 2.72
FEV_1_, L	3.45 ± 0.22 *	4.25 ± 0.32
FEV_1_, % of predicted	93.14 ± 2.78 *	105.80 ± 3.10
Fe_NO_, ppb	67.43 ± 17.98 *	10.71 ± 2.92
PD_20_, mg	0.34 ± 0.07	ND

AA, allergic asthma; BMI, body mass index; Eos, eosinophil; F, female; Fe_NO_, fractional exhaled nitric oxide; FEV_1_, forced expiratory volume in 1 s; HS, healthy subjects; IgE, immunoglobulin E; M, male; ND, not done; PD_20_, provocation dose of methacholine causing a 20% diminish in FEV_1_. Data presented as the mean ± standard error of the mean. Statistical analysis: between investigated groups, Mann–Whitney two-sided U-test. Asterisk (*) indicate *p* < 0.05, compared with the HS group

### Profiling in eosinophil subtypes by NGS

NGS analysis was performed in eosinophil subtypes isolated from 6 patients with AA and 6 HS. Distinct miRNA expression profiles were observed across eosinophil subtypes and study groups. To visualize the overall data structure and sample relationships, we performed multivariate analysis using all 137 differentially expressed miRNAs identified by NGS across study groups and eosinophil subtypes.

**Fig. 2:**
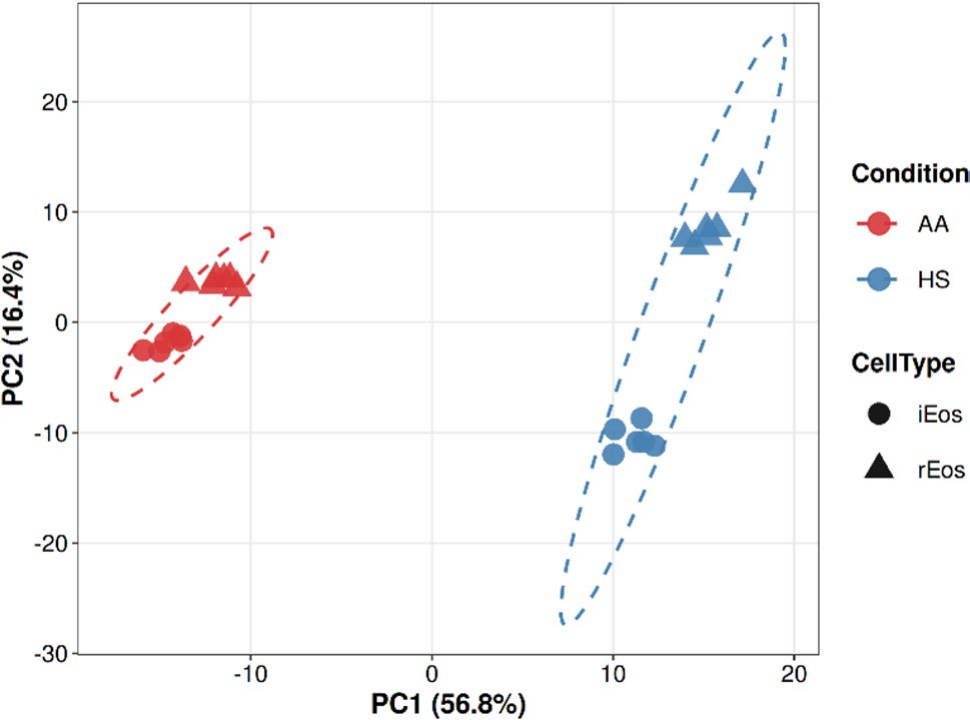
PCA based on the expression of all 137 differentially expressed miRNAs identified by NGS. The scatter plot visualizes the variation between asthma and healthy eosinophil subtypes. The X-axis separates samples by condition status (AA vs HS), while the Y-axis distinguishes cell types (iEos vs rEos). AA n = 6, HS n = 6. AA, allergic asthma; HS, healthy subject; iEos, inflammatory eosinophils; PC, principal component; PCA, principal Component Analysis; rEos, lung-resident eosinophils

PCA revealed a clear segregation of samples into 4 distinct clusters (Fig. 2). The first principal component (PC1), explaining 56.8% of the total variance, clearly discriminated between condition status (AA vs HS). PC2, accounting for 16.4% of the variance, separated the eosinophil subtypes (iEos vs rEos), confirming their distinct miRNA profiles. Pairwise PERMANOVA confirmed significant differences in overall miRNA expression profiles among all four groups, with all six comparisons remaining significant after Benjamini–Hochberg correction (adjusted *p* = 0.006–0.031; Supplementary Table S3). The effect sizes for the comparisons between iEos and rEos within AA and HS were lower (R^2^ = 0.611 and 0.657, respectively; adjusted *p* = 0.031 for both) than those for AA versus HS within iEos and rEos (R^2^ = 0.854 and 0.771, respectively; adjusted *p* = 0.006 for both). This pattern indicates that clinical condition accounted for more variation in the overall miRNA profiles than eosinophil subtype within the AA group and is consistent with AA iEos and AA rEos being more similar to each other than the corresponding eosinophil subtypes across AA and HS. No significant difference in multivariate dispersion was detected among the four groups (PERMDISP: F = 0.316, *p* = 0.852), indicating that the PERMANOVA findings were unlikely to be driven by unequal within-group dispersion.

**Fig. 3:**
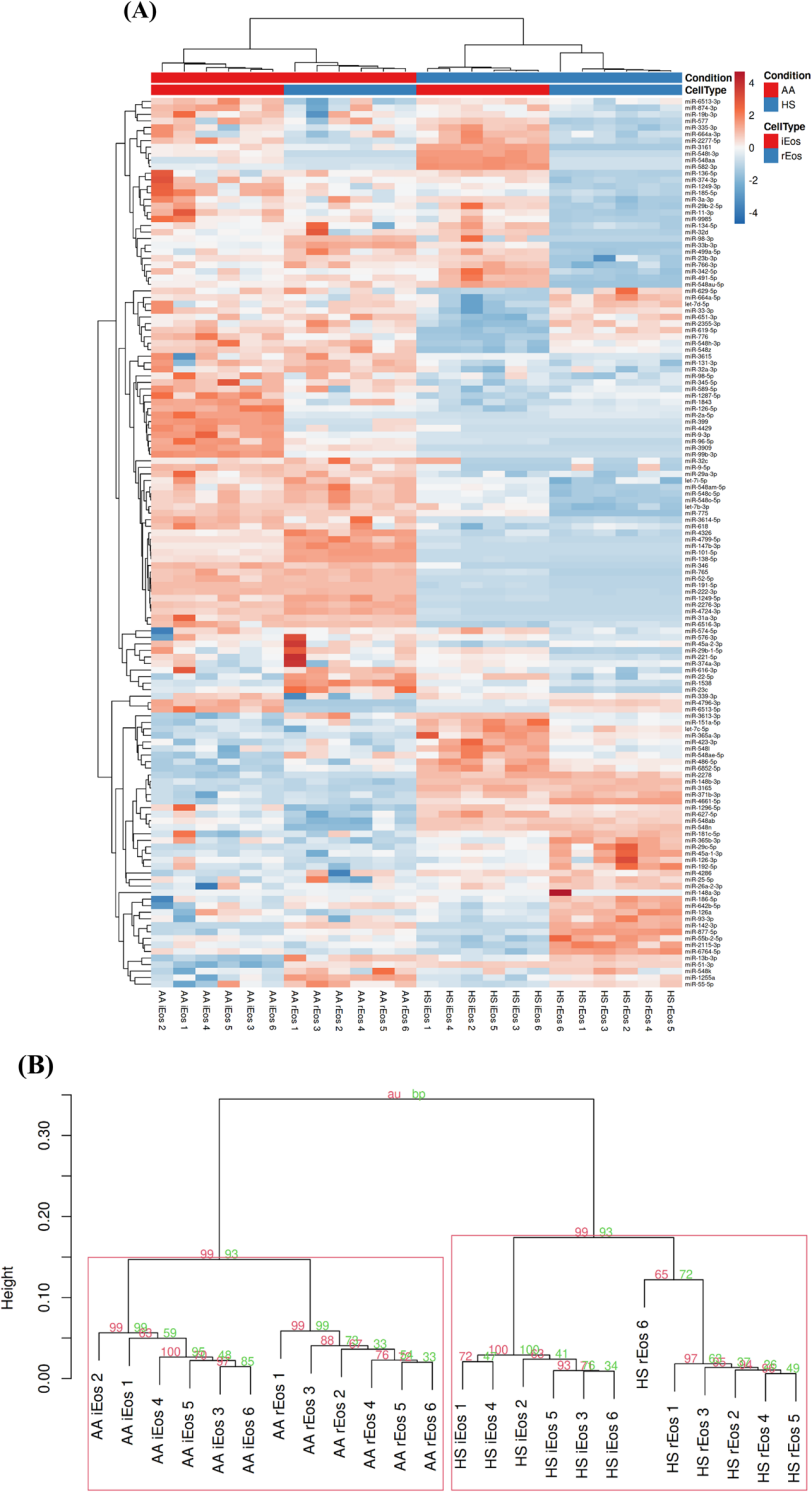
Hierarchical clustering analysis of differentially expressed miRNAs. **A** Unsupervised hierarchical clustering heatmap based on the expression of all 137 differentially expressed miRNAs identified by NGS. Columns represent individual samples, and rows represent individual miRNAs. **B** Multiscale bootstrap resampling dendrogram displaying statistical confidence for hierarchical clustering. Values at nodes indicate AU (red) and BP (green) values (%). Red rectangular boxes highlight main statistically significant clusters with AU ≥ 95%. AA n = 6, HS n = 6. AA, allergic asthma; AU, approximately unbiased; BP, bootstrap probability; HS, healthy subject; iEos, inflammatory eosinophils; rEos, lung-resident eosinophils

Following PCA, unsupervised hierarchical clustering heatmap analysis showed that samples primarily clustered by condition status (AA vs HS), with secondary sub-clustering based on cell type (iEos and rEos) (Fig. 3A). To evaluate the statistical robustness of these groupings, multiscale bootstrap resampling was performed (Fig. 3B). AU *p*-value analysis confirmed high statistical support for both the main condition branches (AU = 99% for AA and HS clusters, indicated by the red rectangles) as well as for the secondary sub-clusters separating iEos and rEos cell types within each condition (AU = 99%).

**Fig. 4:**
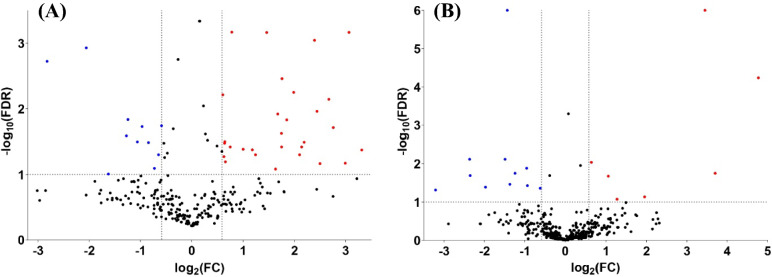
Volcano plots of differentially expressed miRNAs between iEos and rEos. **A** Comparison within the HS group. **B** Comparison within the AA group. The x-axis represents log_2_(FC) and the y-axis represents –log_10_(FDR). The horizontal dashed line indicates the significance threshold (FDR < 0.10; –log_10_ = 1.0), and the vertical dashed lines indicate the fold change threshold (|log_2_(FC)|≥ 0.585, corresponding to a 1.5-fold change). Red dots denote upregulated and blue dots denote downregulated miRNAs in iEos compared with rEos, whereas black dots correspond to non-significant miRNAs. AA, allergic asthma; FC, fold change; FDR, false discovery rate; HS, healthy subject; iEos, inflammatory eosinophil; rEos, lung-resident eosinophil

To further characterize the miRNA differences between eosinophil subtypes observed in the multivariate analysis, we examined the specific miRNAs differentially expressed in each group. In HS, comparison between iEos and rEos revealed differential miRNA expression patterns, as illustrated in Fig. 4A. 40 miRNAs were differentially expressed, of which 11 were downregulated and 29 were upregulated in HS iEos compared with rEos (FDR < 0.10; |log2(FC)|≥ 0.585; Supplementary Table S4). Within the AA group, comparison between iEos and rEos also demonstrated a distinct miRNA signature (Fig. 4B). 18 miRNAs were differentially expressed, including 11 downregulated and 7 upregulated in AA iEos compared with rEos (FDR < 0.10; |log2(FC)|≥ 0.585; Supplementary Table S5).

**Fig. 5:**
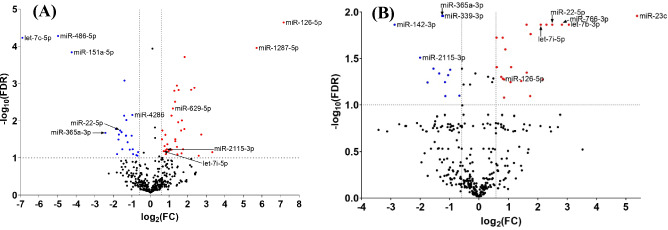
Volcano plots of differentially expressed miRNAs in eosinophil subtypes from AA patients compared with HS. **A** Comparison between AA iEos and HS iEos. **B** Comparison between AA rEos and HS rEos. The x-axis represents log_2_(FC) and the y-axis represents –log_10_(FDR). The horizontal dashed line indicates the significance threshold (FDR < 0.10; –log_10_ = 1.0), and the vertical dashed lines indicate the fold change threshold (|log_2_(FC)|≥ 0.585, corresponding to a 1.5-fold change). Red dots denote miRNAs upregulated in AA compared with HS, while blue dots denote downregulated miRNAs, whereas black dots correspond to non-significant miRNAs. Labeled miRNAs indicate those selected for subsequent qPCR validation. AA, allergic asthma; FC, fold change; FDR, false discovery rate; HS, healthy subjects; iEos, inflammatory eosinophils; qPCR, quantitative polymerase chain reaction; rEos, lung-resident eosinophils

When comparing AA and HS within each eosinophil subtype, substantial differences in miRNA expression were detected. In AA iEos, compared with HS iEos, 62 miRNAs were differentially expressed (26 downregulated, 36 upregulated; FDR < 0.10; |log2(FC)|≥ 0.585; Supplementary Table S6), while in AA rEos, compared with HS rEos, 35 miRNAs showed differential expression (12 downregulated, 23 upregulated; FDR < 0.10; |log2(FC)|≥ 0.585; Supplementary Table S7). The overall distribution of these changes is illustrated in Fig. 5A and B, respectively, and comprehensive lists of all differentially expressed miRNAs identified by NGS are provided in Supplementary Tables S5 and S6.

### Validation of selected miRNAs by qPCR

For qPCR validation, a subset of miRNAs was selected from the NGS results based on statistical significance (FDR < 0.10) and the magnitude of expression change within each eosinophil subtype. Some miRNAs overlapped between the two subtypes, while others were unique to a single subtype. Both upregulated and downregulated miRNAs were included to represent the two directions of regulation observed in the NGS data.

In total, 16 miRNAs were selected for validation. Their expression was assessed by qPCR in both eosinophil subtypes (iEos and rEos) and their corresponding exosomes. The validation cohort included 10 AA and 11 HS samples for cellular eosinophils, and 6 AA and 6 HS samples for eosinophil-derived exosomes. Summary results for these miRNAs are presented in Tables 2 and 3, which combine NGS findings with validation data obtained by qPCR.

**Table 2:** Fold changes and statistical differences of selected miRNAs in AA iEos vs HS iEos and in AA iEos exosomes vs HS iEos exosomes, identified by NGS and validated by qPCR

	NGS			qPCR			
	AA iEos (n = 6) vs HS iEos (n = 6)			AA iEos (n = 10) vs HS iEos (n = 11)		AA iEos exo (n = 6) vs HS iEos exo (n = 6)	
miRNA	Log_2_ FC	FDR	*P*-value	Log_2_ FC	*P*-value	Log_2_ FC	*P*-value
hsa-miR-126-5p	7.17	2.29E − 05	2.70E − 06	1.36	0.01	− 1.90	0.31
hsa-miR-486-5p	− 4.97	5.31E − 05	6.40E − 06	− 1.52	0.01	− 0.10	0.69
hsa-let-7c-5p	− 6.89	5.94E − 05	7.30E − 06	− 0.51	0.05	− 3.23	0.05
hsa-miR-1287-5p	5.71	1.11E − 04	1.39E − 05	0.89	0.01	− 3.48	0.05
hsa-miR-142-3p	0.10	1.16E − 04	1.48E − 05	0.86	0.05	0.27	0.63
hsa-miR-151a-5p	− 4.24	1.46E − 04	1.90E − 05	− 0.72	0.05	2.57	0.13
hsa-miR-629-5p	1.21	4.71E−03	7.17E−04	0.15	0.69	− 0.06	0.81
hsa-miR-4286	− 0.98	7.04E−03	1.09E−03	0.47	0.38	− 0.48	0.63
hsa-miR-22-5p	− 1.63	1.84E−02	3.31E−03	− 0.16	0.03	4.00	0.03
hsa-miR-365a-3p	− 2.42	2.14E−02	4.09E−03	− 1.43	0.02	NA	NA
hsa-miR-766	1.43	5.06E−02	1.16E−02	0.18	0.84	NA	NA
hsa-miR-2115-3p	0.88	5.85E−02	1.36E−02	0.29	0.36	− 3.57	0.25
hsa-let-7i-5p	0.71	6.62E−02	1.66E−02	0.75	0.20	0.55	0.56
hsa-miR-23c	− 0.22	6.86E−01	7.09E−01	− 0.35	0.37	2.71	0.05
hsa-miR-339-3p	0.15	6.33E−01	7.02E−01	− 0.37	0.22	NA	NA
hsa-let-7b-3p	0.86	4.98E−02	1.50E−01	− 0.45	0.35	4.06	0.03

AA, allergic asthma; Exo, exosome; FDR, false discovery rate; HS, healthy subjects; iEos, inflammatory eosinophil; log_2_ FC, log_2_ fold change; NA, not available; NGS, next-generation sequencing; qPCR, quantitative polymerase chain reaction

**Table 3:** Fold changes and statistical differences of selected miRNAs in AA rEos vs HS rEos and in AA rEos exosomes vs HS rEos exosomes, identified by NGS and validated by qPCR

	NGS			qPCR			
	AA rEos (n = 6) vs HS rEos (n = 6)			AA rEos (n = 10) vs HS rEos (n = 11)		AA rEos exo (n = 6) vs HS rEos exo (n = 6)	
miRNA	Log_2_ FC	FDR	*P*-value	Log_2_ FC	*P*-value	Log_2_ FC	*P*-value
hsa-miR-23c	5.38	1.10E−02	9.20E−05	1.21	0.05	3.00	0.05
hsa-miR-365a-3p	− 1.22	1.10E−02	1.01E−04	− 1.37	0.008	6.30	0.05
hsa-miR-339-3p	− 1.25	1.10E−02	9.00E−05	− 1.45	0.05	5.93	0.05
hsa-let-7b-3p	3.06	1.37E−02	3.71E−04	0.16	0.55	4.94	0.03
hsa-let-7i-5p	2.10	1.37E−02	2.68E−04	1.394	0.22	− 4.11	0.05
hsa-miR-142-3p	− 2.87	1.37E−02	3.93E−04	− 0.20	0.99	2.30	0.13
hsa-miR-22-5p	2.49	1.37E−02	3.32E−04	0.89	0.05	4.72	0.03
hsa-miR-766-3p	2.82	1.37E−02	4.18E−04	− 0.27	0.22	2.83	0.03
hsa-miR-2115-3p	− 2.00	3.10E−02	1.52E−03	− 1.09	0.05	3.55	0.05
hsa-miR-126-5p	0.81	5.24E−02	4.63E−03	− 0.93	0.11	4.23	0.05
hsa-miR-1287-5p	− 0.04	9.23E−01	9.39E−01	− 0.11	0.72	4.20	0.03
hsa-miR-151a-5p	− 0.13	8.95E−01	9.18E−01	0.23	0.56	3.03	0.03
hsa-miR-629-5p	− 0.19	7.89E−01	8.65E−01	− 0.23	0.69	0.85	0.63
hsa-miR-4286	− 0.66	5.13E−01	7.07E−01	− 0.11	0.81	1.60	0.31
hsa-miR-486-5p	− 1.43	2.48E−01	5.03E−01	0.03	0.69	2.25	0.30
hsa-let-7c-5p	− 3.42	5.59E−02	1.85E−01	0.01	0.94	3.41	0.05

AA, allergic asthma; Exo, exosome; FDR, false discovery rate; HS, healthy subjects; log_2_ FC, log_2_ fold change; NGS, next-generation sequencing; qPCR, quantitative polymerase chain reaction; rEos, lung-resident eosinophil

**Fig. 6:**
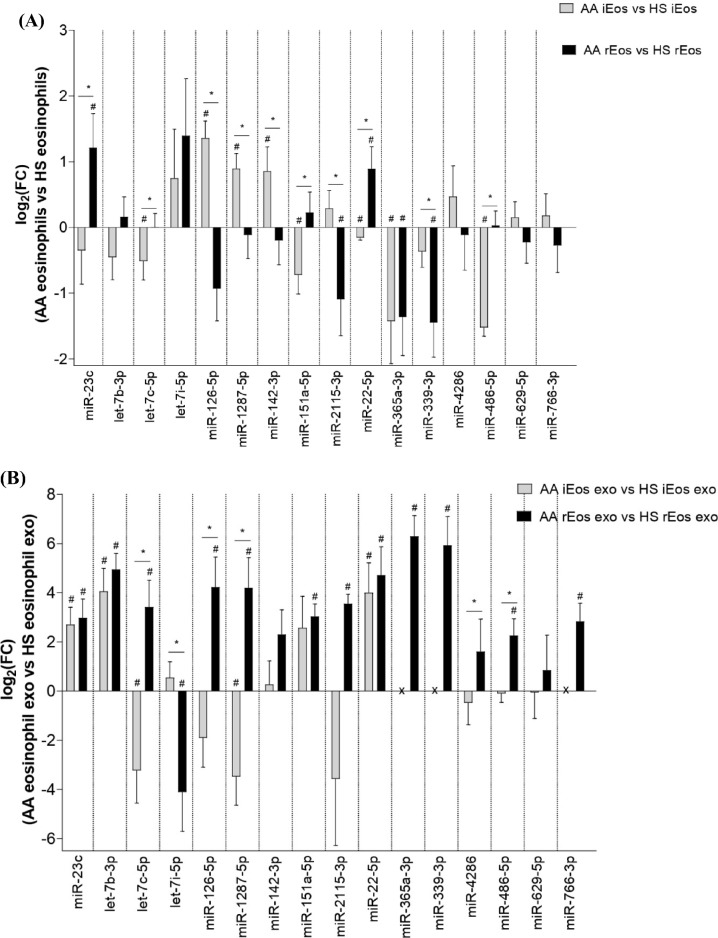
Fold change of miRNA expression in **A** cellular eosinophils and **B** their exosomes. Data represents comparisons of AA iEos vs HS iEos and AA rEos vs HS rEos. Hash marks (#) indicate *p* < 0.05 for comparisons between AA and HS within each eosinophil subtype, while asterisks (*) indicate *p* < 0.05 for comparisons between AA iEos and AA rEos. Crosses (x) in (B) indicate missing data. Sample sizes: **A** AA n = 10, HS n = 11; **B** AA n = 6, HS n = 6. AA, allergic asthma; HS, healthy subjects; iEos, inflammatory eosinophils; rEos, lung-resident eosinophils

A total of 16 selected miRNAs were validated in eosinophil subtypes from AA patients and compared with the corresponding subtypes from HS (Fig. 6A). Among these miRNAs, miR-23c and miR-22-5p were significantly upregulated in AA rEos compared with HS rEos, whereas miR-2115-3p, miR-365a-3p, and miR-339-3p were downregulated (*p* < 0.05). In AA iEos compared with HS iEos, miR-126-5p, miR-1287-5p, and miR-142-3p were upregulated, while let-7c-5p, miR-151a-5p, miR-22-5p, miR-365a-3p, and miR-486-5p were downregulated in AA iEos (*p* < 0.05). When comparing eosinophil subtypes within the AA group, the expression of miR-23c, let-7c-5p, miR-151a-5p, miR-22-5p, and miR-486-5p was higher in rEos, whereas miR-126-5p, miR-1287-5p, miR-142-3p, miR-2115-3p, and miR-339-3p was higher in iEos (p < 0.05; subtype comparisons within AA group are indicated by asterisks in Fig. 6A). The remaining validated miRNAs (let-7b-3p, let-7i-5p, miR-4286, miR-629-5p, and miR-766-3p) did not show significant differences between AA and HS or between eosinophil subtypes (*p* > 0.05).

Additionally, the expression of selected miRNAs was analyzed in eosinophil-derived exosomes from AA and HS groups (Fig. 6B). Several miRNAs, miR-23c, let-7b-3p, and miR-22-5p were significantly upregulated in exosomes derived from both AA iEos and rEos compared with their HS eosinophil subtypes (*p* < 0.05). In AA rEos exosomes, the expression of let-7c-5p, miR-126-5p, miR-1287-5p, miR-151a-5p, miR-2115-3p, miR-365a-3p, miR-339-3p, miR-486-5p, and miR-766-3p was significantly higher, whereas let-7i-5p was downregulated compared with HS rEos exosomes (*p* < 0.05). In AA iEos exosomes, let-7c-5p and miR-1287-5p showed lower expression compared with HS iEos exosomes (*p* < 0.05). Additionally, when comparing eosinophil subtypes within the AA group, let-7c-5p, miR-126-5p, miR-1287-5p, miR-4286, and miR-486-5p were more highly expressed in rEos-derived exosomes, whereas let-7i-5p was more highly expressed in iEos-derived exosomes (*p* < 0.05; subtype comparisons are indicated by asterisks in Fig. 6B).

**Fig. 7:**
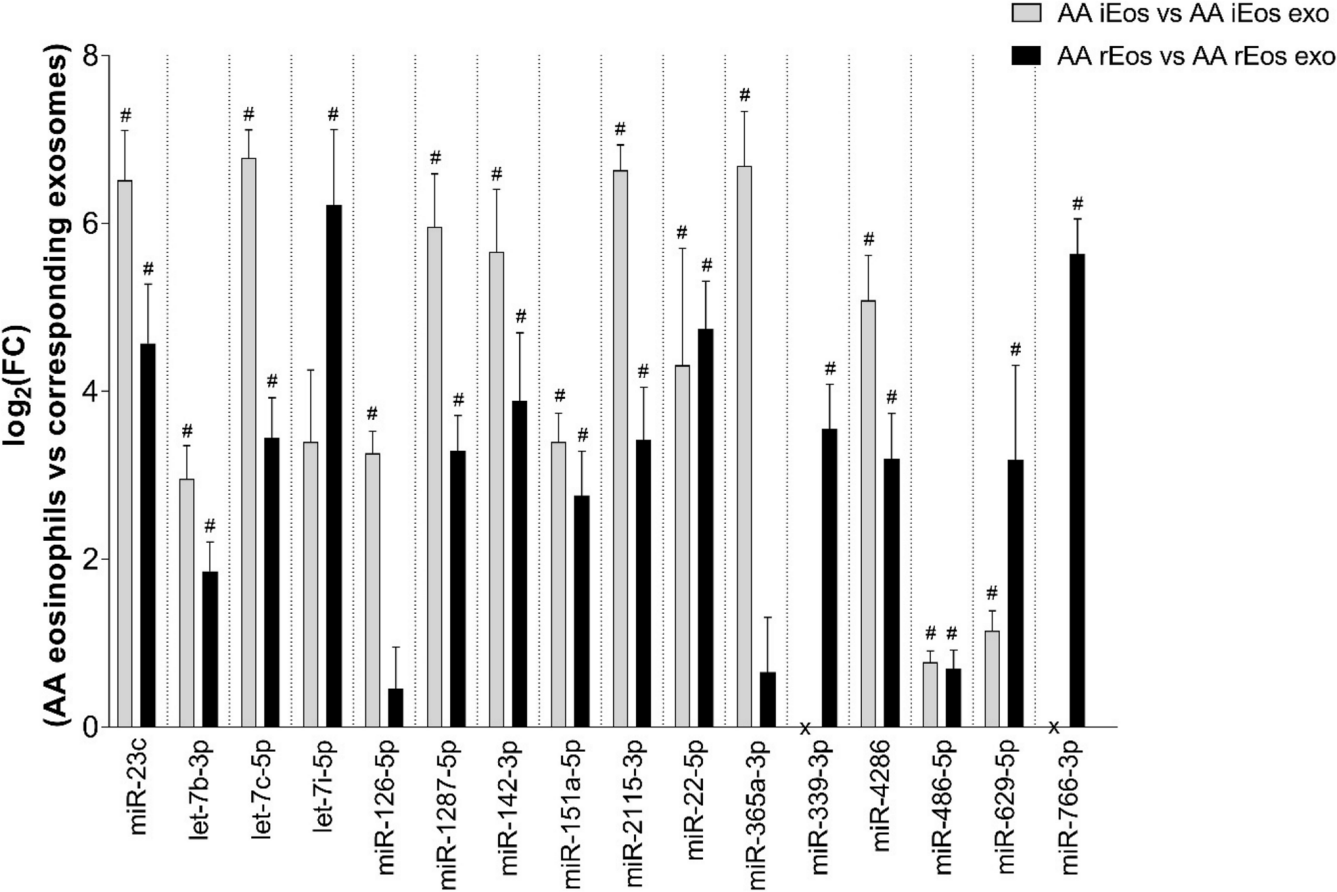
Differential miRNA expression between eosinophils and their corresponding exosomes in patients with AA. Hash marks (#) indicate *p* < 0.05 for comparisons between eosinophils and their respective exosomes. Crosses (x) indicate missing data. AA n = 16, HS n = 17. AA, allergic asthma; Exo, exosomes; FC, fold change; iEos, inflammatory eosinophils; rEos, lung-resident eosinophils

Differences in miRNA expression between eosinophils and their corresponding exosomes from AA patients were examined (Fig. 7). Most analyzed miRNAs showed higher expression in eosinophils compared with their exosomal counterparts. Specifically, miR-23c, let-7b-3p, let-7c-5p, miR-1287-5p, miR-142-3p, miR-151a-5p, miR-2115-3p, miR-22-5p, miR-4286, miR-486-5p, and miR-629-5p had higher expression in both AA iEos and AA rEos compared with their respective exosomes (*p* < 0.05). In addition, let-7i-5p, miR-339-3p, and miR-766-3p showed higher expression in AA rEos compared with their exosomes, whereas miR-126-5p and miR-365a-3p were highly expressed in AA iEos than in AA iEos exosomes (*p* < 0.05).

## Discussions

Increasing evidence indicates that miRNAs and their transfer through exosomes are key modulators of immune activation and tissue remodeling in asthma [28–31]. Yet, miRNA expression profiles specific to distinct eosinophil subtypes and subtype-derived exosomes remain largely unexplored, limiting our understanding of eosinophil-derived regulatory networks in asthma. To address this gap, we performed an integrated analysis of miRNA expression in iEos and rEos and their corresponding exosomes from patients with AA and HS, revealing eosinophil subtype-specific miRNA signatures potentially involved in asthma pathogenesis. NGS analysis identified 62 differentially expressed miRNAs in iEos and 35 in rEos from AA patients compared with their corresponding eosinophil subtypes in the HS group. Of the 16 miRNAs selected for validation, 14 were confirmed to be differentially expressed in either eosinophil subtypes or subtype-derived exosomes between AA and HS. In addition to identifying unique miRNA signatures that distinguish AA iEos and rEos from their HS counterparts, we also observed eosinophil subtype-specific differences within the AA group itself, suggesting functional specialization between iEos and rEos. These findings provide molecular evidence that iEos and rEos represent functionally distinct subtypes rather than different activation states, each contributing uniquely to asthma pathogenesis.

To our knowledge, this is the first study to show that distinct miRNA profiles in circulating eosinophil subtypes and subtype-derived exosomes can discriminate asthma from health, highlighting the biological distinctiveness of eosinophil subtypes and their potential as cell-specific biomarkers. When comparing miRNA expression patterns between eosinophil subtypes and their corresponding exosomes in AA relative to HS, several miRNAs displayed opposite expression trends. This pattern suggests that eosinophils selectively package specific miRNAs into exosomes rather than passively reflecting their intracellular content. Such selective export may fine-tune the signaling potential of eosinophil-derived exosomes and shape intercellular communication within the airway microenvironment. Interestingly, in AA, miRNA enrichment was more pronounced in rEos-derived exosomes than in those from iEos—12 miRNAs were significantly increased in rEos exosomes compared with only 3 in iEos exosomes (both relative to their respective HS controls). This may indicate that rEos are more actively engaged in exosomal communication during asthma, potentially reflecting their role in modulating the airway microenvironment and attempting to restore immune balance during chronic inflammation. Finally, our observation that intracellular miRNA expression was generally higher than in corresponding exosomes in AA suggests that only a limited fraction of the miRNA pool is exported, supporting the notion of an active and selective miRNA export mechanism rather than passive release [32]. A few miRNAs deviated from this pattern (let-7i-5p in iEos, miR-126-5p and miR-365a-3p in rEos), exhibiting comparable levels in cells and exosomes.

To better understand the potential biological relevance of the observed miRNA expression changes, we integrated our findings with evidence from previous studies. Among our analyzed miRNAs, miR-23c expression was upregulated in rEos and in exosomes from both rEos and iEos from AA patients. To date, respiratory research has reported increased miR-23c expression only in endothelial cells from patients with chronic obstructive pulmonary disease [33]. Moreover, miR-23c directly targets *glycogen synthase kinase-3β* (*GSK3β*) [34], a regulator of NF-κB signaling in airway inflammation [35]. Inhibition of *GSK3β* reduces NF-κB phosphorylation and attenuates Th2-driven airway inflammation [36]; thus, elevated miR-23c expression observed in rEos may represent a regulatory attempt aimed at limiting NF-κB activation and inflammatory signaling. Increased miR-23c in iEos-derived exosomes may further indicate a paracrine mechanism to modulate excessive airway inflammation.

Beyond miR-23c, differential expression of several let-7 family members (let-7b-3p, let-7c-5p, and let-7i-5p) highlights the complex post-transcriptional regulation of eosinophil function in asthma. These miRNAs share a conserved seed sequence (nucleotides 2–8) allowing overlapping target recognition [37], although variations outside the seed region (particularly at positions 13–16) confer distinct biological functions [38]. Importantly, the role of the let-7 family in allergic airway inflammation appears context-dependent. In vivo, systemic inhibition of let-7 miRNAs in murine asthma models reduced Th2 cytokine production, eosinophil accumulation, and airway hyperreactivity [39], whereas in vitro studies revealed that IL-13 mRNA is a direct target of let-7 miRNAs in Th2 cells, implying cell-specific effects [40].

Increased let-7b-3p expression in both AA iEos and rEos-derived exosomes, as observed in our study, may influence the regulation of airway inflammation. Previous studies have shown that let-7b expression increases in the lung following allergen challenge and is associated with airway epithelial and smooth muscle cell responses [40]. Moreover, let-7b can regulate innate immune responses by directly targeting *interferon beta (IFN-β)* mRNA [41]. Since type I interferons suppress Th2 differentiation and cytokine secretion by inhibiting GATA3 transcription factor [42], elevated let-7b-3p expression in eosinophil-derived exosomes may therefore attenuate IFN-β signaling, thereby promoting a Th2-skewed inflammatory environment characteristic of AA.

Similarly, let-7i-5p, known as a pro-inflammatory miRNA in airway epithelium, enhances inflammation by targeting *DUSP1* and activating MAPK signaling, leading to increased cytokine release and airway hyperresponsiveness [43]. It also promotes pathogenic fibroblast migration and upregulates TGF-β1 and MMP2 through upregulation of *VIM*, *MMP1*, yet directly suppresses IL-13, indicating dual and context-dependent roles in inflammation and remodeling [44]. The downregulation of let-7i-5p in rEos-derived exosomes from AA patients may represent a cell-specific compensatory response aimed at limiting MAPK-mediated pro-inflammatory signaling within the asthmatic airway.

In contrast, let-7c-5p exhibited an opposite expression pattern—upregulated in rEos exosomes but reduced in iEos and their exosomes in AA, suggesting cell-type-specific regulation. As let-7c suppresses IL-1β, IL-6, and TNF-α and is upregulated by anti-inflammatory agents such as dexamethasone and non-steroidal anti-inflammatory drugs, it is linked to the resolution of inflammation [45]. Elevated let-7c levels have also been reported in infants with asthma [46], while reduced expression correlates with increased eosinophilia in asthmatic patients [47]. Thus, higher rEos-derived exosomal let-7c-5p expression may contribute to inflammatory resolution, whereas its reduction in iEos may reflect a loss of regulatory control.

Another miRNA implicated in epithelial remodeling, miR-1287-5p, suppresses *SNAI1* and *HMGB1*, thereby reducing IL-6, IL-8, and TNF-α release and inhibiting epithelial–mesenchymal transition [48]. In our study, miR-1287-5p expression was increased in iEos and rEos-derived exosomes but decreased in iEos-derived exosomes from AA patients, suggesting a limited paracrine anti-inflammatory capacity of iEos compared to the regulatory signaling of rEos. Given that eosinophils can promote EMT and fibrosis through TGF-β1 and IL-1β secretion [49], the enrichment of miR-1287-5p in rEos exosomes may serve as a protective mechanism counteracting airway remodeling.

While miR-1287-5p appears linked to epithelial repair, another miRNA, miR-365a-3p, primarily modulates cytokine-mediated inflammatory signaling. miR-365a-3p suppresses IL-17–induced cytokine production (IL-6, IL-8) in airway epithelial cells and macrophages by targeting *β-arrestin-2* (*ARRB2*), thereby attenuating IL-17-driven inflammation [50]. Although IL-17 is mainly associated with neutrophilic responses, it can also contribute to Th2-associated eosinophilic inflammation in asthma [51–53]. In our study, miR-365a-3p expression was downregulated in iEos and rEos from AA patients, suggesting enhanced IL-17 signaling and eosinophil activation, while its upregulation in rEos-derived exosomes may reflect a compensatory anti-inflammatory mechanism limiting airway inflammation and remodeling.

We also identified altered expression of miR-339-3p. Elevated circulating miR-339-3p levels correlate with reduced lung function (lower FVC% predicted) in asthmatic children [54] and poor corticosteroid responsiveness [55], likely due to targeting of *NR3C1*, which encodes the glucocorticoid receptor, thereby limiting steroid sensitivity and sustaining Th2 inflammation [56]. In contrast, reduced miR-339-3p expression in chronic rhinosinusitis with nasal polyps and its genetic association with lower disease risk suggest a protective role [57]. Functionally, miR-339-3p promotes apoptosis and inhibits nasal epithelial proliferation via PI3K–Akt pathway modulation [57, 58]. In our study, miR-339-3p expression was decreased in rEos but increased in their exosomes from AA, suggesting active secretion as a regulatory mechanism to limit airway inflammation. Together, these findings indicate a dual role for miR-339-3p as a negative regulator of type 2 inflammation and remodeling, where intracellular loss and exosomal enrichment may reflect dysregulated inflammatory control in asthma.

Several less-characterized miRNAs, including miR-151a-5p and miR-2115-3p, also showed altered expression patterns suggestive of roles in airway inflammation. miR-151a-5p has been associated with a reduced risk of incident asthma, particularly in individuals with high vitamin D levels [59]. In our study, miR-151a-5p was upregulated in rEos-derived exosomes but downregulated in iEos from AA patients, suggesting potential involvement in anti-inflammatory signaling and intercellular communication within the asthmatic airway. miR-2115-3p expression was increased in rEos-derived exosomes but decreased in rEos from AA patients. Although scarcely investigated, this miRNA has been reported to be expressed in peripheral blood cells, including eosinophils, neutrophils, and monocytes, in healthy individuals, while elevated levels in the blood of patients with non-small-cell lung cancer were associated with systemic immune cell activation [60], supporting a possible pro-inflammatory role in eosinophil-mediated airway responses.

Following the miRNAs with potential anti-inflammatory effects, we next focused on those with pro-inflammatory potential. One such miRNA is miR-126-5p, which was upregulated in iEos and rEos-derived exosomes from AA patients. Previous studies identified miR-126-5p as a pro-inflammatory miRNA that promotes allergic airway inflammation by targeting *HIPK2*, a negative regulator of NF-κB signaling, which plays a central role in mediating inflammatory responses [61]. Elevated miR-126-5p enhances NF-κB activation via increased IκBα and p65 phosphorylation, and upregulates mediators such as IL-5, TNF-α, ECP, LTC4, and PGD2, while its inhibition reduces inflammation and restores anti-inflammatory cytokines IL-2 and IFN-γ expression (62). Thus, increased miR-126-5p expression in iEos may indicate NF-κB–driven inflammatory activation, whereas its enrichment in rEos-derived exosomes may indicate a paracrine mechanism sustaining airway inflammation.

A similar pattern was observed for miR-142-3p. Eosinophilia and elevated IgE are hallmarks of Th2-type inflammation in asthma, and eosinophils express both low- (CD23) and high-affinity (FcεRI) IgE receptors [63]. In mast cells, miR-142-3p enhances FcεRI-dependent degranulation by promoting Ca^2^⁺ mobilization and regulating the actin cytoskeleton via *lipoma-preferred partner* (*LPP*) targeting [64]; eosinophil degranulation relies on similar Ca^2^⁺-dependent actin remodeling [65]. In our study, miR-142-3p was upregulated in iEos from AA patients, suggesting heightened degranulation capacity possibly through these actin-related pathways. Moreover, miR-142-3p can promote airway smooth muscle proliferation [66] by targeting *Adenomatous polyposis coli* (*APC*) and activating WNT/β-catenin signaling [67]. Taken together, elevated miR-142-3p in AA iEos may contribute to both inflammatory amplification and airway remodeling.

Another miRNA with pro-inflammatory potential identified in our study was miR-22-5p, which was increased in rEos, iEos, and rEos-derived exosomes from AA patients. Although miR-22-3p is well known for anti-inflammatory effects [68, 69], the role of miR-22-5p remains less defined. Elevated miR-22-5p levels have been associated with increased TLR4 and NF-κB protein expression, suggesting that it may normally suppress a negative regulator of the TLR4/NF-κB pathway and that its upregulation enhances pro-inflammatory signaling [70]. Clinically, miR-22-5p distinguishes asthma subtypes, being higher in patients with non-steroidal anti-inflammatory drug–exacerbated respiratory disease than in AA, and correlates positively with blood eosinophil counts [71].

Similarly, miR-486-5p showed an expression pattern suggestive of pro-inflammatory and pro-remodeling activity. In our study, miR-486-5p was increased in rEos-derived exosomes but decreased in iEos from AA patients. Although its role in asthma remains controversial, studies have reported both downregulation of miR-486-5p in eosinophils [9] and elevated levels in asthma patients [72, 73], suggesting context-dependent regulation. Functionally, miR-486-5p targets *aquaporin-5* (*AQP5*), a protective factor that limits airway inflammation and remodeling. Suppression of miR-486-5p or overexpression of *AQP5* alleviates AA by reducing cytokine secretion, goblet-cell proliferation, and smooth-muscle remodeling, whereas miR-486-5p overexpression restores these pathological features [73, 74]. Additionally, miR-486-5p targets *PTEN* and *FoxO1*, thereby activating PI3K/Akt signaling, which enhances cell survival and proliferation—processes central to airway remodeling. Moreover, it can suppress Smad1/2/4, attenuating TGF-β signaling, a key regulator of fibrosis and epithelial–mesenchymal transition [75, 76]. Collectively, increased exosomal miR-486-5p from eosinophils may promote inflammation and airway remodeling through *AQP5* suppression and PI3K/Akt–TGF-β pathway activation in recipient cells.

Finally, circulating miR-766-3p has been linked to frequent exacerbations in children with asthma and adults with chronic obstructive pulmonary disease, reflecting heightened airway inflammation [77]. Single-cell RNA sequencing further identified miR-766-3p among exacerbation-associated miRNAs broadly expressed in airway epithelial and immune cells [78]. In our study, miR-766-3p was upregulated exclusively in rEos-derived exosomes from AA patients, suggesting selective export and a paracrine role in sustaining airway inflammation.

## Limitations

This study has several limitations. The primary aim was to characterize miRNA expression changes in eosinophil subtypes and their exosomes in AA; therefore, functional studies exploring downstream effects were beyond its scope. Nevertheless, several additional methodological factors should also be considered. First, the sample size was relatively small, which may limit the statistical power and generalizability of the findings. This limitation was particularly relevant to the NGS cohort, which comprised six subjects per group and may therefore have increased uncertainty in the estimated miRNA expression differences. Accordingly, the NGS findings should be interpreted as exploratory and hypothesis-generating, with greater emphasis placed on candidates subsequently evaluated by qPCR. Second, given the influence of the tissue microenvironment on eosinophil plasticity [79, 80], our analysis was limited to circulating eosinophil subtypes, and the identified miRNA patterns may differ from those of eosinophils residing in target tissues, where expression likely adapts to local conditions in vivo*.* Third, not all differentially expressed miRNAs identified by NGS were included in the validation step and confirmed by qPCR. Fourth, our cohort included only patients with non-severe AA who had not used inhaled corticosteroids for at least one month before enrollment; therefore, the identified eosinophil subtype-specific miRNA profiles may not be directly generalizable to moderate or severe asthma. More severe disease might be associated with more pronounced or qualitatively different miRNA alterations rather than a uniform amplification of the signatures observed in our cohort, as eosinophil and circulating miRNA profiles have previously been associated with asthma severity and distinct clinical phenotype [9, 15]. However, the direction and magnitude of these differences cannot be predicted from the present data. Their interpretation would also be complicated by treatment exposure, as circulating miRNAs have been associated with clinical response to inhaled corticosteroids [81], while anti-IL-5 pathway therapies can alter eosinophil abundance, activation, and subtype distribution, preferentially reducing iEos and resulting in a relatively greater proportion of rEos (7, 82). Therefore, miRNA profiles in treated moderate or severe asthma could reflect both underlying disease activity and treatment-related attenuation or redistribution of subtype-specific signals. Longitudinal studies evaluating eosinophil subtype-specific miRNA expression before and after treatment would be required to distinguish severity-related from therapy-related effects. Finally, the study did not include experimental validation of miRNA–target interactions or downstream functional effects in eosinophils. Thus, the proposed mechanisms remain hypothetical and require further in vitro and in vivo confirmation.

## Conclusions

This study identified distinct miRNA expression signatures in iEos and rEos as well as in their exosomes from patients with AA, clearly distinguishing them from those of HS. Moreover, within the AA group, iEos and rEos exhibited distinct miRNA expression patterns, indicating subtype-specific regulation. These findings provide molecular evidence that eosinophil subtypes are functionally specialized, each characterized by a unique miRNA profile that likely reflects their differential contributions to asthma pathogenesis.

In AA, rEos-derived exosomes exhibited broader miRNA enrichment than those from iEos when compared with HS, suggesting a more active involvement of rEos in exosomal communication under inflammatory conditions. Furthermore, the rEos miRNA pattern pointed toward a regulatory or compensatory role in modulating airway inflammation and maintaining tissue balance, whereas iEos predominantly expressed pro-inflammatory miRNAs. However, these interpretations should be viewed with caution, as some exceptions were observed and direct functional validation was not performed.

Overall, our results highlight the selective export of miRNAs as a potential mechanism through which eosinophils contribute to airway inflammation and remodeling. Future experimental and longitudinal studies are warranted to confirm the mechanistic significance of these findings and to evaluate the potential of eosinophil- and exosome-derived miRNAs as biomarkers or therapeutic targets in asthma.

## Informed consent

Informed consent was obtained from all subjects involved in the study.

## Supplementary Information

Below is the link to the electronic supplementary material.

Supplementary Material 1

## Data Availability

Data are available from the corresponding author on reasonable request.
