# Supplementary material for: Distinct miRNA expression patterns in circulating eosinophil subtypes and their subtype-derived exosomes in allergic asthma
Source: Inflamm Res. 2026 Aug 18;75(1):192. doi: 10.1007/s00011-026-02348-w (PMC13481468; doi:10.1007/s00011-026-02348-w)
Supplement: Supplementary file 1 [file 11_2026_2348_MOESM1_ESM.docx]

**Table S1.** Inclusion and exclusion criteria.

| **Inclusion criteria** | |
| --- | --- |
| Allergic asthma patients (n = 22) | Healthy subjects (n = 21) |
| - Allergic asthma symptoms for ≥ 1 year - A non-severe course of the disease - Free of inhaled steroids for ≥ 1 month - Positive skin prick test to *Dermatophagoides pteronyssinus* (house dust mite) allergen - Positive methacholine challenge test | - No chronic respiratory or other diseases - No clinical allergy symptoms - Negative skin prick test |
| **Exclusion criteria** | |
| - Age < 18 years - Active airway infection ≤ 1 month prior to study - Other chronic airway diseases - Other clinically significant non-controlled disease - Active airway infection ≤ 1 month prior to study - Use of oral steroids ≤ 1 month prior to study - Smoking - Pregnancy, breastfeeding | |

**Table S2.** Investigations at visits.

| **Study visits and performed procedures** | |
| --- | --- |
| Visit 1 | Visit 2 |
| Inclusion/exclusion criteria  Written informed consent  Spirometry  Metacholine challange test  Skin prick test | Complete blood count  Total IgE  Spirometry  Fe_NO_ measurement |

Fe_NO_ – fractional exhaled nitric oxide; IgE – immunoglobulin E.

**Table S3.** Pairwise PERMANOVA comparisons of overall miRNA expression profiles among AA iEos, AA rEos, HS iEos, and HS rEos. Pseudo-F denotes the PERMANOVA test statistic, and R² represents the proportion of multivariate variation explained by group membership. Adjusted p values were calculated using the Benjamini–Hochberg procedure.

| **Comparison** | **Design** | **Pseudo-F** | **R^2^** | **p-value** | **FDR-adjusted p-value** |
| --- | --- | --- | --- | --- | --- |
| AA iEos vs AA rEos | Paired | 15.74 | 0.611 | 0.031 | 0.031 |
| AA iEos vs HS iEos | Unpaired | 58.34 | 0.854 | 0.003 | 0.006 |
| AA iEos vs HS rEos | Unpaired | 40.07 | 0.800 | 0.002 | 0.006 |
| AA rEos vs HS iEos | Unpaired | 52.58 | 0.840 | 0.002 | 0.006 |
| AA rEos vs HS rEos | Unpaired | 33.72 | 0.771 | 0.004 | 0.006 |
| HS iEos vs HS rEos | Paired | 19.12 | 0.657 | 0.031 | 0.31 |

AA – allergic asthma; FDR – false discovery rate; HS – healthy subjects; iEos – inflammatory eosinophils; rEos – lung-resident eosinophils.

**Table S4.** Fold changes and statistical differences of miRNAs in HS iEos vs rEos, identified by NGS.

|  | **NGS** | | |
| --- | --- | --- | --- |
|  | **HS iEos vs rEos (n = 6)** | | |
| **miRNA** | **Log_2_ FC** | **FDR** | **P-value** |
| hsa-miR-3613-3p | 0.78 | 6.76E-04 | 3.48E-04 |
| hsa-miR-766-3p | 3.07 | 6.80E-04 | 3.53E-04 |
| hsa-miR-577 | 1.46 | 6.84E-04 | 3.58E-04 |
| hsa-miR-23c | 2.39 | 8.98E-04 | 4.74E-04 |
| hsa-miR-1287-5p | -2.06 | 1.17E-03 | 6.24E-04 |
| hsa-miR-2115-3p | -2.82 | 1.89E-03 | 1.02E-03 |
| hsa-miR-22-5p | 1.76 | 3.47E-03 | 1.89E-03 |
| hsa-miR-3a-3p | 1.99 | 5.62E-03 | 3.09E-03 |
| hsa-miR-221-5p | 0.60 | 6.12E-03 | 3.39E-03 |
| hsa-miR-29b-1-5p | 2.67 | 7.17E-03 | 4.00E-03 |
| hsa-miR-136-5p | 2.44 | 1.10E-02 | 6.20E-03 |
| hsa-miR-32d | 1.67 | 1.20E-02 | 6.87E-03 |
| hsa-miR-93-3p | -1.24 | 1.46E-02 | 8.40E-03 |
| hsa-miR-335-3p | 1.85 | 1.48E-02 | 8.57E-03 |
| hsa-miR-55-5p | -0.59 | 1.82E-02 | 1.06E-02 |
| hsa-miR-181c-5p | -0.97 | 1.87E-02 | 1.10E-02 |
| hsa-miR-151a-5p | 2.76 | 1.94E-02 | 1.15E-02 |
| hsa-miR-1249-3p | 1.75 | 2.38E-02 | 1.43E-02 |
| hsa-miR-126a | -1.27 | 2.59E-02 | 1.58E-02 |
| hsa-miR-874-3p | 0.64 | 3.15E-02 | 1.95E-02 |
| hsa-miR-651-3p | -1.06 | 3.17E-02 | 1.97E-02 |
| hsa-let-7b-3p | 2.19 | 3.21E-02 | 2.01E-02 |
| hsa-miR-2355-3p | -0.84 | 3.25E-02 | 2.05E-02 |
| hsa-miR-19b-3p | 0.64 | 3.33E-02 | 2.13E-02 |
| hsa-miR-11-3p | 1.75 | 3.79E-02 | 2.49E-02 |
| hsa-miR-486-5p | 2.14 | 3.79E-02 | 2.47E-02 |
| hsa-miR-576-3p | 0.75 | 3.79E-02 | 2.45E-02 |
| hsa-let-7i-5p | 1.00 | 4.11E-02 | 2.72E-02 |
| hsa-miR-548l | 1.18 | 4.19E-02 | 2.78E-02 |
| hsa-let-7c-5p | 3.32 | 4.21E-02 | 2.81E-02 |
| hsa-miR-134-5p | 1.24 | 4.97E-02 | 3.43E-02 |
| hsa-miR-186-5p | -0.65 | 4.97E-02 | 3.41E-02 |
| hsa-miR-2277-5p | 2.10 | 4.97E-02 | 3.42E-02 |
| hsa-miR-374a-3p | 0.62 | 5.32E-02 | 3.70E-02 |
| hsa-miR-185-5p | 0.65 | 6.39E-02 | 4.49E-02 |
| hsa-miR-491-5p | 2.99 | 6.71E-02 | 4.75E-02 |
| hsa-miR-499a-5p | 2.50 | 6.80E-02 | 4.84E-02 |
| hsa-miR-45a-2-3p | -0.73 | 8.03E-02 | 5.75E-02 |
| hsa-miR-664a-3p | 1.63 | 8.24E-02 | 5.94E-02 |
| hsa-miR-664a-5p | -1.63 | 9.79E-02 | 7.10E-02 |

FDR – false discovery rate; HS – healthy subjects; iEos – inflammatory eosinophil; log_2_ FC – log_2_ fold change; NGS – next-generation sequencing; rEos – lung-resident eosinophil.

**Table S5.** Fold changes and statistical differences of miRNAs in AA iEos vs rEos, identified by NGS.

|  | **NGS** | | |
| --- | --- | --- | --- |
|  | **AA iEos vs rEos (n = 6)** | | |
| **miRNA** | **Log_2_ FC** | **FDR** | **P-value** |
| hsa-miR-98-3p | -1.43 | 1.00E-06 | 1.00E-06 |
| hsa-miR-99b-3p | 3.45 | 1.00E-06 | 1.00E-06 |
| hsa-miR-126-5p | 4.77 | 5.80E-05 | 4.00E-06 |
| hsa-miR-1538 | -1.49 | 7.65E-03 | 5.35E-04 |
| hsa-miR-22-5p | -2.37 | 7.65E-03 | 5.28E-04 |
| hsa-miR-126-3p | 0.65 | 9.15E-03 | 6.60E-04 |
| hsa-miR-548ae-5p | -0.95 | 1.31E-02 | 1.00E-03 |
| hsa-miR-101-5p | -1.24 | 1.78E-02 | 1.44E-03 |
| hsa-miR-1287-5p | 3.70 | 1.78E-02 | 1.42E-03 |
| hsa-miR-33b-3p | -2.35 | 2.04E-02 | 1.73E-03 |
| hsa-miR-3909 | 1.06 | 2.11E-02 | 1.85E-03 |
| hsa-miR-138-5p | -1.37 | 3.45E-02 | 3.09E-03 |
| hsa-miR-4724-3p | -0.94 | 3.72E-02 | 3.42E-03 |
| hsa-miR-147b-3p | -1.97 | 4.09E-02 | 3.84E-03 |
| hsa-miR-6852-5p | -0.62 | 4.36E-02 | 4.19E-03 |
| hsa-miR-23c | -3.21 | 4.86E-02 | 4.78E-03 |
| hsa-miR-6513-5p | 1.95 | 7.34E-02 | 7.38E-03 |
| hsa-miR-185-5p | 1.27 | 8.45E-02 | 8.68E-03 |

AA – allergic asthma; FDR – false discovery rate; iEos – inflammatory eosinophil; log_2_ FC – log_2_ fold change; NGS – next-generation sequencing; rEos – lung-resident eosinophil.

**Table S6.** Fold changes and statistical differences of miRNAs in AA iEos vs HS iEos and in AA iEos exosomes vs HS iEos exosomes, identified by NGS and validated by qPCR.

|  | **NGS** | | | **qPCR** | | | |
| --- | --- | --- | --- | --- | --- | --- | --- |
|  | **AA iEos (n = 6)**  **vs HS iEos (n = 6)** | | | **AA iEos (n = 10) vs HS iEos (n = 11)** | | **AA iEos exo (n = 6) vs HS iEos exo (n = 6)** | |
| **miRNA** | **Log_2_ FC** | **FDR** | **P-value** | **Log_2_ FC** | **P-value** | **Log_2_ FC** | **P-value** |
| **hsa-miR-126-5p** | **7.17** | **2.29E-05** | **2.70E-06** | **1.36** | **0.01** | **-1.90** | **0.31** |
| **hsa-miR-486-5p** | **-4.97** | **5.31E-05** | **6.40E-06** | **-1.52** | **0.01** | **-0.10** | **0.69** |
| **hsa-let-7c-5p** | **-6.89** | **5.94E-05** | **7.30E-06** | **-0.51** | **0.05** | **-3.23** | **0.05** |
| **hsa-miR-1287-5p** | **5.71** | **1.11E-04** | **1.39E-05** | **0.89** | **0.01** | **-3.48** | **0.05** |
| **hsa-miR-151a-5p** | **-4.24** | **1.46E-04** | **1.90E-05** | **-0.72** | **0.05** | **2.57** | **0.13** |
| hsa-miR-4799-5p | 1.84 | 1.95E-04 | 2.59E-05 | NA | NA | NA | NA |
| hsa-miR-6852-5p | -1.40 | 8.38E-04 | 1.13E-04 | NA | NA | NA | NA |
| hsa-miR-3614-5p | 1.42 | 1.15E-03 | 1.58E-04 | NA | NA | NA | NA |
| hsa-miR-4724-3p | 2.37 | 1.31E-03 | 1.84E-04 | NA | NA | NA | NA |
| hsa-miR-399 | 2.19 | 1.48E-03 | 2.11E-04 | NA | NA | NA | NA |
| hsa-miR-2276-3p | 1.52 | 1.50E-03 | 2.17E-04 | NA | NA | NA | NA |
| hsa-miR-147b-3p | 1.29 | 1.58E-03 | 2.32E-04 | NA | NA | NA | NA |
| hsa-miR-619-5p | 1.33 | 3.11E-03 | 4.66E-04 | NA | NA | NA | NA |
| **hsa-miR-629-5p** | **1.21** | **4.71E-03** | **7.17E-04** | **0.15** | **0.69** | **-0.06** | **0.81** |
| **hsa-miR-4286** | **-0.98** | **7.04E-03** | **1.09E-03** | **0.47** | **0.38** | **-0.48** | **0.63** |
| hsa-miR-3165 | -1.41 | 7.37E-03 | 1.19E-03 | NA | NA | NA | NA |
| hsa-miR-548h-3p | 1.13 | 7.37E-03 | 1.20E-03 | NA | NA | NA | NA |
| hsa-miR-548z | 1.13 | 7.37E-03 | 1.20E-03 | NA | NA | NA | NA |
| hsa-miR-29c-5p | -1.30 | 9.64E-03 | 1.59E-03 | NA | NA | NA | NA |
| hsa-miR-138-5p | 1.69 | 9.91E-03 | 1.66E-03 | NA | NA | NA | NA |
| hsa-miR-1843 | 1.50 | 1.10E-02 | 1.87E-03 | NA | NA | NA | NA |
| hsa-miR-548n | -1.86 | 1.67E-02 | 2.92E-03 | NA | NA | NA | NA |
| hsa-miR-52-5p | 1.82 | 1.68E-02 | 2.97E-03 | NA | NA | NA | NA |
| **hsa-miR-22-5p** | **-1.63** | **1.84E-02** | **3.31E-03** | **-0.16** | **0.03** | **4.00** | **0.03** |
| hsa-miR-6764-5p | 0.64 | 1.84E-02 | 3.39E-03 | NA | NA | NA | NA |
| hsa-miR-33-3p | 1.65 | 1.84E-02 | 3.39E-03 | NA | NA | NA | NA |
| hsa-miR-548aa | -1.55 | 2.04E-02 | 3.86E-03 | NA | NA | NA | NA |
| hsa-miR-548t-3p | -1.55 | 2.04E-02 | 3.86E-03 | NA | NA | NA | NA |
| **hsa-miR-365a-3p** | **-2.42** | **2.14E-02** | **4.09E-03** | **-1.43** | **0.02** | **NA** | **NA** |
| hsa-miR-4429 | 2.73 | 2.38E-02 | 4.64E-03 | NA | NA | NA | NA |
| hsa-miR-3161 | -1.70 | 2.38E-02 | 4.68E-03 | NA | NA | NA | NA |
| hsa-miR-589-5p | 0.81 | 2.41E-02 | 4.81E-03 | NA | NA | NA | NA |
| hsa-miR-548ae-5p | -1.31 | 2.52E-02 | 5.08E-03 | NA | NA | NA | NA |
| hsa-miR-627-5p | -1.01 | 2.54E-02 | 5.18E-03 | NA | NA | NA | NA |
| hsa-miR-3613-3p | -1.32 | 2.55E-02 | 5.26E-03 | NA | NA | NA | NA |
| hsa-miR-765 | 1.34 | 3.16E-02 | 6.67E-03 | NA | NA | NA | NA |
| hsa-miR-9-5p | 0.63 | 3.17E-02 | 6.78E-03 | NA | NA | NA | NA |
| hsa-miR-2278 | -1.72 | 3.23E-02 | 6.99E-03 | NA | NA | NA | NA |
| hsa-miR-4796-3p | 1.34 | 3.58E-02 | 7.83E-03 | NA | NA | NA | NA |
| hsa-miR-423-3p | -1.23 | 3.79E-02 | 8.39E-03 | NA | NA | NA | NA |
| hsa-miR-45a-2-3p | 0.91 | 4.30E-02 | 9.62E-03 | NA | NA | NA | NA |
| hsa-miR-2355-3p | 0.76 | 4.83E-02 | 1.09E-02 | NA | NA | NA | NA |
| **hsa-miR-766** | **1.43** | **5.06E-02** | **1.16E-02** | **0.18** | **0.84** | **NA** | **NA** |
| **hsa-miR-2115-3p** | **0.88** | **5.85E-02** | **1.36E-02** | **0.29** | **0.36** | **-3.57** | **0.25** |
| hsa-miR-4661-5p | -0.95 | 5.85E-02 | 1.37E-02 | NA | NA | NA | NA |
| hsa-miR-548au-5p | -1.51 | 5.85E-02 | 1.38E-02 | NA | NA | NA | NA |
| hsa-miR-1249-5p | 1.75 | 5.85E-02 | 1.39E-02 | NA | NA | NA | NA |
| hsa-miR-51-3p | -1.12 | 6.02E-02 | 1.45E-02 | NA | NA | NA | NA |
| hsa-miR-618 | 1.13 | 6.02E-02 | 1.46E-02 | NA | NA | NA | NA |
| hsa-miR-346 | 0.82 | 6.27E-02 | 1.54E-02 | NA | NA | NA | NA |
| hsa-miR-775 | 0.98 | 6.55E-02 | 1.63E-02 | NA | NA | NA | NA |
| **hsa-let-7i-5p** | **0.71** | **6.62E-02** | **1.66E-02** | **0.75** | **0.20** | **0.55** | **0.56** |
| hsa-miR-198-5p | 3.33 | 7.02E-02 | 1.78E-02 | NA | NA | NA | NA |
| hsa-miR-191-5p | -0.67 | 7.02E-02 | 1.79E-02 | NA | NA | NA | NA |
| hsa-miR-185-5p | 1.68 | 7.54E-02 | 1.96E-02 | NA | NA | NA | NA |
| hsa-miR-766-3p | -0.94 | 7.90E-02 | 2.08E-02 | NA | NA | NA | NA |
| hsa-miR-32c | -1.80 | 7.91E-02 | 2.10E-02 | NA | NA | NA | NA |
| hsa-miR-13b-3p | 0.83 | 7.93E-02 | 2.12E-02 | NA | NA | NA | NA |
| hsa-miR-9-3p | 1.45 | 8.04E-02 | 2.17E-02 | NA | NA | NA | NA |
| hsa-miR-548ab | -0.76 | 8.43E-02 | 2.30E-02 | NA | NA | NA | NA |
| hsa-miR-45a-1-3p | -0.70 | 8.75E-02 | 2.42E-02 | NA | NA | NA | NA |
| hsa-miR-96-5p | 2.59 | 8.75E-02 | 2.43E-02 | NA | NA | NA | NA |
| **hsa-miR-23c** | **-0.22** | **6.86E-01** | **7.09E-01** | **-0.35** | **0.37** | **2.71** | **0.05** |
| **hsa-miR-339-3p** | **0.15** | **6.33E-01** | **7.02E-01** | **NA** | **NA** | **NA** | **NA** |
| **hsa-let-7b-3p** | **0.86** | **4.98E-02** | **1.50E-01** | **-0.45** | **0.35** | **4.06** | **0.03** |
| **hsa-miR-142-3p** | **0.10** | **1.16E-04** | **1.48E-05** | **0.86** | **0.05** | **0.27** | **0.63** |

AA – allergic asthma; Exo – exosome; FDR – false discovery rate; HS – healthy subjects; iEos – inflammatory eosinophil; log_2_ FC – log_2_ fold change; NA – not available; NGS – next-generation sequencing; qPCR – quantitative polymerase chain reaction.

**Table S7.** Fold changes and statistical differences of miRNAs in AA rEos vs HS rEos and in AA rEos exosomes vs HS rEos exosomes, identified by NGS and validated by qPCR.

|  | **NGS** | | | **qPCR** | | | |
| --- | --- | --- | --- | --- | --- | --- | --- |
|  | **AA rEos (n = 6) vs HS rEos (n = 6)** | | | **AA rEos (n = 10) vs HS rEos (n = 11)** | | **AA rEos exo (n = 6) vs HS rEos exo (n = 6)** | |
| **miRNA** | **Log_2_ FC** | **FDR** | **P-value** | **Log_2_ FC** | **P-value** | **Log_2_ FC** | **P-value** |
| **hsa-miR-23c** | **5.38** | **1.10E-02** | **9.20E-05** | **1.21** | **0.05** | **3.00** | **0.05** |
| **hsa-miR-365a-3p** | **-1.22** | **1.10E-02** | **1.01E-04** | **-1.37** | **0.008** | **6.30** | **0.05** |
| **hsa-miR-339-3p** | **-1.25** | **1.10E-02** | **9.00E-05** | **-1.45** | **0.05** | **5.93** | **0.05** |
| **hsa-let-7b-3p** | **3.06** | **1.37E-02** | **3.71E-04** | **0.16** | **0.55** | **4.94** | **0.03** |
| **hsa-let-7i-5p** | **2.10** | **1.37E-02** | **2.68E-04** | **1.394** | **0.22** | **-4.11** | **0.05** |
| **hsa-miR-142-3p** | **-2.87** | **1.37E-02** | **3.93E-04** | **-0.20** | **0.99** | **2.30** | **0.13** |
| **hsa-miR-22-5p** | **2.49** | **1.37E-02** | **3.32E-04** | **0.89** | **0.05** | **4.72** | **0.03** |
| hsa-miR-491-5p | 2.29 | 1.37E-02 | 2.41E-04 | NA | NA | NA | NA |
| hsa-miR-6516-3p | 1.62 | 1.37E-02 | 3.61E-04 | NA | NA | NA | NA |
| **hsa-miR-766-3p** | **2.82** | **1.37E-02** | **4.18E-04** | **-0.27** | **0.22** | **2.83** | **0.03** |
| hsa-miR-548c-5p | 1.75 | 1.73E-02 | 6.34E-04 | NA | NA | NA | NA |
| hsa-miR-548o-5p | 1.75 | 1.73E-02 | 6.34E-04 | NA | NA | NA | NA |
| hsa-miR-1255a | 0.80 | 1.89E-02 | 8.08E-04 | NA | NA | NA | NA |
| hsa-miR-342-5p | 0.60 | 1.89E-02 | 7.73E-04 | NA | NA | NA | NA |
| hsa-miR-616-3p | 0.89 | 2.53E-02 | 1.16E-03 | NA | NA | NA | NA |
| **hsa-miR-2115-3p** | **-2.00** | **3.10E-02** | **1.52E-03** | **-1.09** | **0.05** | **3.55** | **0.05** |
| hsa-miR-131-3p | 0.61 | 3.92E-02 | 2.16E-03 | NA | NA | NA | NA |
| hsa-miR-29b-2-5p | 1.09 | 3.92E-02 | 2.14E-03 | NA | NA | NA | NA |
| hsa-miR-126a | -1.55 | 4.08E-02 | 2.38E-03 | NA | NA | NA | NA |
| hsa-miR-877-5p | -0.98 | 4.19E-02 | 2.68E-03 | NA | NA | NA | NA |
| hsa-miR-548am-5p | 1.62 | 4.49E-02 | 3.02E-03 | NA | NA | NA | NA |
| hsa-miR-1296-5p | -1.35 | 4.59E-02 | 3.36E-03 | NA | NA | NA | NA |
| hsa-miR-6764-5p | -1.05 | 4.79E-02 | 3.66E-03 | NA | NA | NA | NA |
| hsa-miR-374-3p | 0.75 | 5.00E-02 | 4.12E-03 | NA | NA | NA | NA |
| **hsa-miR-126-5p** | **0.81** | **5.24E-02** | **4.63E-03** | **-0.93** | **0.11** | **4.23** | **0.05** |
| hsa-miR-136-5p | 2.17 | 5.34E-02 | 4.96E-03 | NA | NA | NA | NA |
| hsa-miR-574-5p | 0.85 | 5.34E-02 | 5.05E-03 | NA | NA | NA | NA |
| hsa-miR-11-3p | 1.43 | 5.54E-02 | 5.41E-03 | NA | NA | NA | NA |
| hsa-miR-32a-3p | 1.07 | 5.70E-02 | 6.08E-03 | NA | NA | NA | NA |
| hsa-miR-365b-3p | -1.15 | 5.70E-02 | 6.03E-03 | NA | NA | NA | NA |
| hsa-miR-192-5p | -1.75 | 5.75E-02 | 6.31E-03 | NA | NA | NA | NA |
| hsa-miR-26a-2-3p | -0.66 | 7.97E-02 | 9.48E-03 | NA | NA | NA | NA |
| hsa-miR-371b-3p | -1.14 | 8.05E-02 | 9.92E-03 | NA | NA | NA | NA |
| hsa-miR-9985 | 1.74 | 8.05E-02 | 1.01E-02 | NA | NA | NA | NA |
| hsa-miR-185-5p | 0.85 | 8.35E-02 | 1.07E-02 | NA | NA | NA | NA |
| **hsa-miR-1287-5p** | **-0.04** | **9.23E-01** | **9.39E-01** | **-0.11** | **0.72** | **4.2** | **0.03** |
| **hsa-miR-151a-5p** | **-0.13** | **8.95E-01** | **9.18E-01** | **0.23** | **0.56** | **3.03** | **0.03** |
| **hsa-miR-629-5p** | **-0.19** | **7.89E-01** | **8.65E-01** | **-0.23** | **0.69** | **0.85** | **0.63** |
| **hsa-miR-4286** | **-0.66** | **5.13E-01** | **7.07E-01** | **-0.11** | **0.81** | **1.6** | **0.31** |
| **hsa-miR-486-5p** | **-1.43** | **2.48E-01** | **5.03E-01** | **0.03** | **0.69** | **2.25** | **0.3** |
| **hsa-let-7c-5p** | **-3.42** | **5.59E-02** | **1.85E-01** | **0.01** | **0.94** | **3.41** | **0.05** |

AA – allergic asthma; Exo – exosome; FDR – false discovery rate; HS – healthy subjects; log_2_ FC – log_2_ fold change; NA – not available; NGS – next-generation sequencing; rEos – lung-resident eosinophil; qPCR – quantitative polymerase chain reaction.
